# Does successful urethral calibration rule out significant female urethral stenosis? confronting the confounder- an outcome analysis of successfully treated female urethral strictures

**DOI:** 10.1590/S1677-5538.IBJU.2020.0857

**Published:** 2021-03-05

**Authors:** Sidhartha Kalra, Praanjal Gupta, Lalgudi N. Dorairajan, Manikandan Ramanitharan, Sreerag Kodakkattil Sreenivasan, Sovan Hota

**Affiliations:** 1 JIPMER Department of Urology and Renal Transplantation Puducherry India Department of Urology and Renal Transplantation, JIPMER, Puducherry, India

**Keywords:** Female, Urinary Bladder, Urethral Stricture

## Abstract

**Objective:**

The diagnosis and treatment of female urethral stricture disease (FUSD) are practiced variably due to the scarcity of data on evaluation, variable definitions, and lack of long-term surgical outcomes. FUSD is difficult to rule out solely on the basis of a successful calibration with 14F catheter. In this study, we have tried to characterize the variable clinical presentation of FUSD, the diagnostic utility of calibration, videourodynamic study(VUDS), and urethroscopy in planning surgical management.

**Materials and Methods:**

A retrospective review of records of 16 patients who underwent surgical management of FUSD was analyzed. The clinical history, examination findings, and the results of all the investigations (including uroflowmetry, VUDS findings, urethroscopy) they underwent, the procedures they had undergone, and the follow-up data were studied.

**Results:**

A total of 16 patients underwent surgical management of FUSD. 13 out of 16 patients had successful calibration with 14F catheter on the initial presentation. These 13 patients on VUDS demonstrated significant BOO and had variable stigmata of stricture on urethroscopy. The mean IPSS, flow rate, and PVR at presentation and after urethroplasty were 23.88±4.95, 7.72±4.25mL/s, 117.06±74.46mL and 3.50±3.44, 22.34±4.80mL/s, and 12.50±8.50mL, respectively. (p <0.05). The mean flow rate after endo dilation(17F) (n=12) was 11.4±2.5mL/s while after urethroplasty improved to 20.30±4.19mL/s and was statistically significant(p <0.05).

**Conclusions:**

An adept correlation between clinical assessment, urethroscopy findings, and VUDS is key in objectively identifying FUSD and planning surgical management. A good caliber of the urethra is not sufficient enough to rule out a significant obstruction due to FUSD. Early urethroplasty provides significantly better outcomes in patients who have failed dilation as a treatment.

## INTRODUCTION

Female urethral stricture disease (FUSD) is a rare clinical entity with a broad spectrum of clinical presentation, creating a dilemma regarding surgical management. This problem's true incidence is not apparent due to the under-diagnosis and under-treatment of this complex pathology. However, about 4-13% of all causes of bladder outlet obstruction (BOO) have been attributed to urethral stricture (1, 2). Urethral stricture has been defined as asymptomatic, anatomical narrowing of the urethra based upon a failure of catheterization, urethral calibration, visual inspection, on endoscopy or ‘radiography’ by Osman et al. (3). However, to objectively integrate all these criteria to reach the diagnosis can be a challenge. The etiology of strictures in women may be idiopathic, infective from urethritis, iatrogenic due to prior dilation, difficult catheterization with subsequent fibrosis or urethral or vaginal surgery external trauma. Recurrent dilation in women with persistent lower urinary tract symptoms (LUTS) is a common practice, thereby confounding the diagnosis of true urethral stricture or stenosis. The diagnosis and treatment of FUSD are poorly studied with the scarcity of data on evaluation, variable definitions, and long-term surgical outcomes (4). The exact reason why patients on recurrent urethral dilation and those with successful calibration keep dawdling with voiding LUTS is still a grey area.

Further, in the absence of any standard assessment protocol for FUSD, most urologists rely on a mixed bag of findings on patient's history, physical examination, and investigations. FUSD is difficult to rule out solely based on a successful calibration with 14F. We hypothesized that these patients can be identified earlier with an apt clinical, VUDS, and endoscopic correlation-based diagnostic approach. An early reconstructive procedure in patients with significant BOO due to FUSD who fail endodilation yields better outcomes. We in this study, have tried to characterize the variable clinical presentation of FUSD, the diagnostic utility of calibration, videourodynamic study (VUDS) and urethroscopy in planning surgical management. Along with it, we have studied the outcomes of female urethroplasty in patients who fail urethral endodilation and have tried to correlate the outcomes with the preoperative findings. Finally, we procreate a plausible diagnostic approach for patients likely to benefit from surgical intervention in FUSD.

## MATERIAL AND METHODS

A retrospective review of data of all female patients with voiding LUTS diagnosed with FUSD and who underwent surgical management in the Department of Urology at our tertiary care center from October 2017 to November 2019, collected from an IRB approved database (JIP/IEC/2020/089) was performed. Their demographic features like age and BMI were noted. The clinical history, examination findings, and the results of all the investigations (including uroflowmetry, VUDS findings, urethroscopy) they underwent, the procedures they had undergone, and the follow-up data were recorded.

### Diagnostic protocol for FUSD

**Figure 1:**
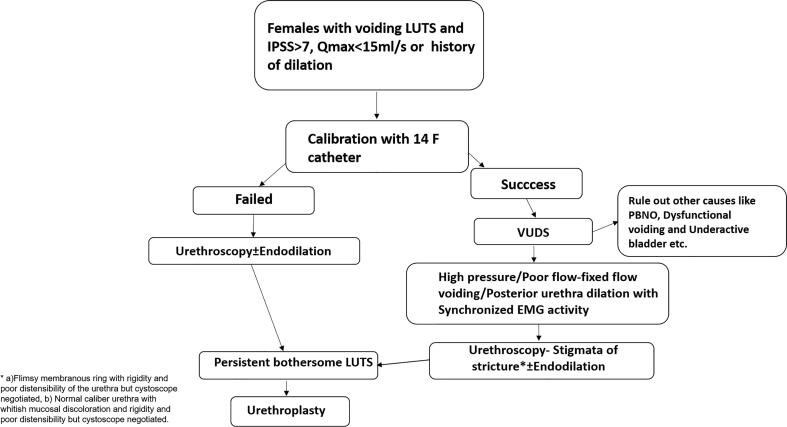
Management protocol for patients suspected with FUSD.

At our institute, we routinely screen for bladder outlet obstruction (BOO) in patients who have the presence of voiding LUTS and have uroflowmetry flow rate <15mL/s or IPSS>7, or a post-void residual urine (PVR)>100mL on ultrasonography or a history of prior urethral dilation with or without improvement in flow. All these subjects are then calibrated with 14F catheter at the outpatient clinic to confirm or rule out the presence of any obvious anatomical obstruction. However, a mild catch or a subjective difficulty in inserting the catheter is not considered as failed calibration. Those patients who fail calibration are subjected to urethroscopy. In contrast, the rest who had successful calibration is further evaluated with VUDS better to characterize their lower urinary tract symptoms (BOO) and rule out detrusor underactivity. On VUDS, after ruling out other causes of BOO such as primary bladder neck obstruction (PBNO) and dysfunctional voiding, the diagnosis of urethral stricture was suspected when there was presence of urethral ballooning proximal to a portion of urethral narrowing along with urodynamic BOO defined as a sustained detrusor contraction of any magnitude with a fixed flow pattern of Qmax <15mL/sec and a synergic sphincter EMG activity. Findings of increased sphincter activity on EMG during voiding with the interrupted flow along with a spinning top deformity of the urethra on fluoroscopy were suggestive of dysfunctional voiding. All patients suspected of having urethral stricture disease on calibration or VUDS underwent urethroscopy, initially with a 7.5F ureteroscope to characterize urethral mucosal abnormality with any obvious obliteration and further, with a 17F cystoscope sheath to assess rigidity and distensibility of the urethra. Three types of findings were noted, 1) Obvious narrowing of the urethral lumen with inability to negotiate the cystoscope, 2) Flimsy membranous ring with rigidity and poor distensibility of the urethra but cystoscope negotiated, 3)Normal caliber urethra with whitish mucosal discoloration and rigidity and poor distensibility but cystoscope negotiated. We considered the last two in the category of “variable stigmata of possible sequelae of urethral stricture” owing to the subjective nature of those findings (Table-1). Poor distensibility and rigidity were observed when there was resistance in negotiating 17F cystoscope associated with mucosal trauma. After the endoscopy, these patients were further assessed to see for improvement in voiding based on clinical symptoms and uroflowmetry (Figure-1). All VUDS, endoscopic assessment, and surgery were performed by a single surgeon to minimize subjective variability in management. After considering patient's preferences and correlation of clinical, urethroscopy and VUDS characteristics the patients underwent urethral dilation or urethroplasty for FUSD with a dorsal onlay technique. Vaginal and buccal mucosal grafts were used.

**Table 1:** Differential Diagnosis of Female urethral stricture disease(FUSD).

Clinical Diagnosis (Voiding LUTS/IPSS>7,Qmax<15mL/s)	FUSD (Failed calibration)	FUSD (Successful calibration)	Dysfunctional voiding/Detrusor Sphincter Dyssynergia	Primary Bladder neck Obstruction(PBNO)	Underactive bladder
**Significant clinical history**	H/o recurrent dilation, instrumentation, recurrent urinary tract infection (UTI), H/O possible improvement in flow after dilation	H/o recurrent dilation, instrumentation, Recurrent UTI, H/O Possible improvement in flow after dilation	**Primary Storage symptoms** Situational Voiding difficulty. Need to ‘deliberately’ relax themselves.	Incomplete emptying with High residue. H/O Acute or Chronic retention	Hesitancy, sensation of incomplete emptying, straining to void H/o prior surgery, Diabetes Mellitus
**Calibration with 14F catheter**	**Failed**	Successful	Successful	Successful	Successful
**VUDS with EMG**	**Urodynamics -Not required** Micturating Cystourethrogram sufficient- Bladder neck funneling with Presence of urethral ballooning proximal to a portion of urethral narrowing.	**Recommended** High pressure/Poor flow(fixed flat flow voiding). Adequate opening of bladder neck with presence of urethral ballooning proximal to a portion of urethral narrowing)with Synchronized Electromyogram (EMG) activity	**Recommended** Moderate to High pressure/Poor flow interrupted spinning top urethra with Desynchronized EMG activity	**Recommended** Moderate to High pressure/Poor flow(fixed flow voiding) with no or inadequate bladder neck opening with Synchronized EMG activity	**Recommended** Low pressure/low flow prolonged voiding time. Straining or fixed flow pattern with opening of bladder neck and synchronised EMG activity
**Urethroscopy (17 F)**	Obvious obliteration and inability to negotiate the scope	Stigmata of stricture*	Normal study	Normal study	Normal Study

* a)Flimsy membranous ring with rigidity and poor distensibility of the urethra but cystoscope negotiated; b) Normal caliber urethra with whitish mucosal discoloration and rigidity and poor distensibility but cystoscope negotiated.

### Our operative technique of urethroplasty

**Figure 2:**
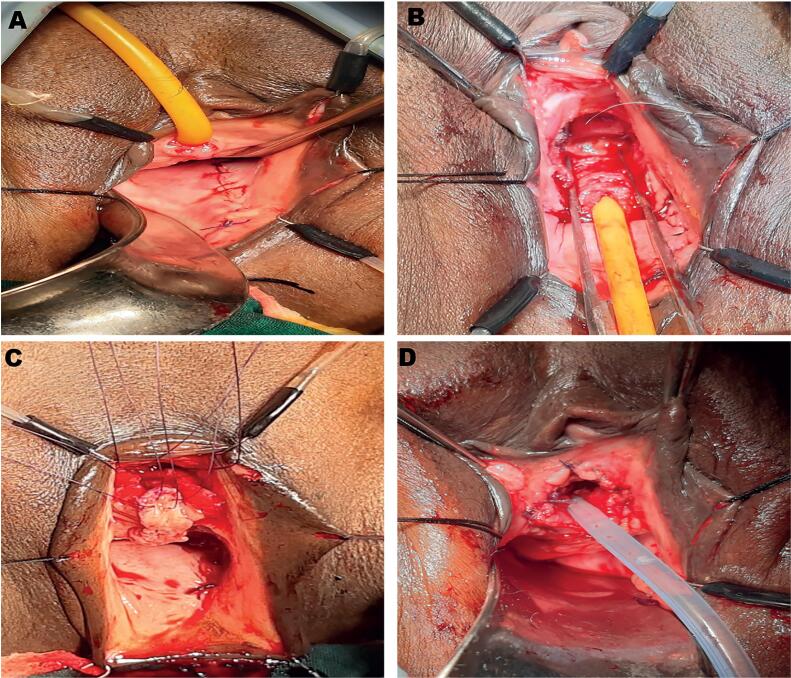
Surgical steps in dorsal onlay graft urethroplasty. 2A- Harvesting of vaginal graft from posterior lateral vaginal wall and closure of harvest site. 2B-Dorsal dissection of urethra from clitoral bed up to the bladder neck after detachment of pubourethral ligament. 2C- Dorsal urethrotomy extending well beyond the stricture and preplaced sutures taken through apex of the laid open urethra and the vaginal graft. 2D- Finally constructed neomeatus.

All patients underwent substitution urethroplasty with a dorsal onlay approach. After placing the patient in lithotomy position perimeatal incision was given dorsally, urethral dissection was done up to the bladder, and urethra was opened dorsally. Dorsal onlay vaginal/buccal graft urethroplasty was done by quilting the graft dorsally with pubourethral fascia and clitoral bed with running suture using Vicryl 3-0 and anastomosing the graft edges with laid open urethral edge using the same type of suture (Figure-2). A 16F silicon Foley catheter was placed at the end of the procedure and retained for three weeks. For patients with post-radiation strictures, the catheter was retained for 6 weeks.

Postoperatively, all patients were followed at 1, 3, and 12 months after urethroplasty or dilation with IPSS score, uroflowmetry, and PVR estimation. Failure was defined as patients with worsening symptoms requiring urethral dilation or secondary surgical intervention or maximum flow rate <15 (Qmax) with/without inability to calibrate the urethra with a 14F catheter. Episodes of incontinence, urinary tract infections, and bothersome LUTS persistence were also recorded in the follow-up period.

## RESULTS

A total of 16 patients underwent surgical management of FUSD between October 2017 to November 2019. The median age of the patients was 52 years (range 40-76 years). The mean duration of symptoms was 5.7 years (4 months-20 years). The mean IPSS, flow rate, and PVR at presentation were 23.88±4.95, 7.72±4.25mL/s, and 117.06±74.46mL, respectively. Three out of 16 patients (18.75%) had history of acute urinary retention in the past. Three patients (18.75%) had presented with acute renal failure. Twelve patients (75%) had a history of prior Hegar urethral dilation at different centers for LUTS. (Table-2)

**Table 2:** Clinical, VUDS, and urethroscopic characteristics of patients with Female urethral stricture.

Characteristic	Sub-category	Number (%), Mean±SD
**Age(Years)**		52.31±10.51
**Site of stricture**	Proximal	2(12)
	Mid	10(63)
	Distal	3(19)
	Panurethral	1(6)
**Etiology**	Idiopathic (H/O recurrent dilation)	12(68.75%)
	Lichen Sclerosis	1(12.5%)
	Radiation	3(18.75%)
**Improvement in voiding following dilation(n=12/16)**	Yes	11(91.67%)
	No	1(8.33%)
**Calibration with 14F foley**	Successful	13 (81.25%)
	Failed	3 (18.75%)
**Urethroscopy(17Fr)**	Nonnegotiable; Obvious obliteration of urethral lumen with the inability to negotiate the scope	4(18.75%)
	Flimsy membranous ring with rigidity and poor distensibility of the urethra but cystoscope negotiated.	6(37.5%)
	Normal caliber urethra with whitish mucosal discoloration and rigidity and poor distensibility, but cystoscope negotiated.	6(43.75%)
**VUDS(n=13)**	**Parameter**	**Value (Mean±SD)**
	Capacity	414±120mL
	Pdet@endfill	6.23±5.36cmH2O
	Compliance	102.66±60.76
	Detrusor overactivity(n)	1
	Qmax	7.23±3.23mL/s
	Pdet@Qmax	69.85±20.49cmH2O
	Flow pattern curve - Flat fixed curve	12
	EMG activity synchronized	13
	Fluoroscopy video in voiding phasebladder neck widening with posterior urethra dilation	13
**Type of reconstruction**	Vaginal graft	13
	Buccal mucosa	3

pdet@Qmax = detrusor pressure at maximum flow; SD = standard deviation; Qmax = Maximum flow rate; VUDS = Video urodynamic study; PVR = post void residual urine

On presentation at our institution, three patients had failed calibration, while 13 had successful calibration with a 14F Foley catheter. These 13 patients on VUDS demonstrated significant BOO with mean pdet@Qmax of 69.85±20.48cmH2O and mean flow rate of 7.23±3.72mL/s (Table-1) (Figure-2).

**Figure 3:**
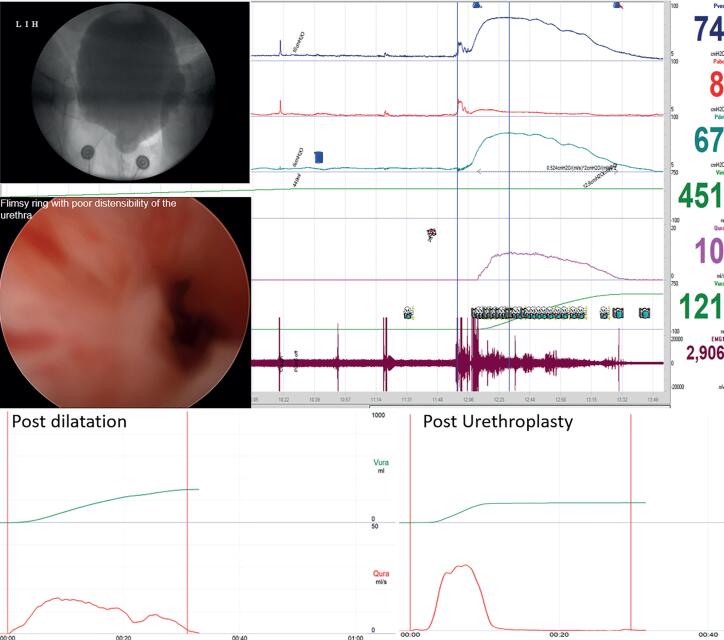
43-year-old lady with IPSS (27) with successful calibration with 14F, showing VUDS and urethroscopic correlation with a pDet@Qmax 67cmH2O, flat fixed flow curve, proximal urethral ballooning, stigmata of stricture as a flimsy ring with poor distensibility of urethra, Qmax after endodilation~14mL/s and after urethroplasty ~30mL/s.

On urethroscopy in 4 (25%) patients, the scope couldn't be negotiated due to tough, dense stricture, while 12 (75%) had variable stigmata of stricture (Table-2). The mean flow rate after endo dilation (17F) (n=12) was 11.4±2.5mL/s while after urethroplasty improved to 20.30±4.19mL/ and was statistically significant (p <0.05). The flow pattern was near normal after urethroplasty in contrast to the flow pattern after endo dilation (Figure-3).

The mean IPSS, flow rate, and PVR after urethroplasty(n=16) were 3.50±3.44, 22.34±4.80mL/s, and 12.50±8.50mL respectively and were statistically significant (p <0.05). Further, a comparison of various parameters based on the success of calibration is given in Table-3.

**Table 3:** Comparison of various parameters among FUSD with failed and successful calibration(14F) group.

Parameter	Failed calibration(n=3)	Successful calibration with VUDS and urethroscopy correlation suggestive of stricture (n=13)
Mean IPSS(Preop)	27.33±2.0	23.08±5.12
Mean IPSS (Postop)	2.67±2.5	3.69±3
Mean Duration of symptoms(years)	7.83±10.6	5.25±3.8
Recurrent dilation	2(66.7%)	10(83.3%)
Qmax (Preop) (mL/S)	4.6±2.4	8.44±4.32
Qmax (Postop)(mL/S)	26.1±4.5	21.46±4.5
Urethroscopy(17F)	Obvious narrowing with scope non negotiable	Stigmata (n= 12) Narrowing with scope non negotiable (n=1)
PVR(Preop)(mL)	200±130	97.9±44.6
PVR(Postop)(mL)	9±8	13.3±8.7

**Qmax** = maximum flow rate; **SD** = standard deviation; **PVR** = post void residual urine; **IPSS** = International prostate symptom score

A total of three buccal grafts were used, one each for the case of panurethral stricture, radiation stricture, and lichen sclerosis, while in the rest, 13 vaginal grafts were used. One patient had reported transient stress incontinence after urethroplasty, which resolved after a month. None of the patients reported persistence of bothersome LUTS or recurrence of the stricture in the subsequent follow-up of up to 12 months.

## DISCUSSION

Female urethral stricture is an uncommonly diagnosed clinical entity that can profoundly impact the quality of life (5). Among women having LUTS, 2.7-8% are attributed to bladder outlet obstruction, and further a meager 4-13% of those with bladder outlet obstruction will have apparent anatomical stenosis (2). A large number of these women with possible anatomical obstruction with LUTS routinely undergo urethral instrumentation to temporarily alleviate the symptoms, thereby casting a shadow on the true prevalence and understanding of this disease. Recurrent empirical treatment without proper diagnostic evaluation and definitive surgical intervention can further aggravate this problem. A major hurdle has been a lack of clearly defined diagnostic criteria of this rare entity and explicit comparison of various treatment outcomes (6).

Although limited evidence is available to portray the causes of stricture, contemporary studies have identified a few contributory factors. Most cases of female stricture disease are likely iatrogenic or traumatic, depending on whether they originate from prolonged catheterization, pelvic radiation, childbirth, pelvic fracture, or surgical repair for diverticulum, fistula, or incontinence, recurrent empirical dilation for LUTS (7). In our study, 12 patients (75%) had a history of prior recurrent dilation done at various centers for their voiding LUTS. Whether these were the treatment for their disease or cause, it is a matter of debate. However, 11 of these patients (91.67%) did have marginal improvement on dilation. A systematic review stated that stricture etiology was identifiable in 53% of women; of these cases, most were either idiopathic (49%) or iatrogenic (39%), with the remainder either traumatic (7%) or inflammatory (6%) (3).

Female urethral stricture is an entity with the contentious dogma of diagnosis. Women typically present with frequency and urgency symptoms, with additional complaints of dysuria, hesitancy, dribbling, incontinence, and recurrent urinary tract infections (8). Rare reports of urinary retention, renal failure, hydronephrosis, and pyelonephritis have been attributed to FUSD (9). Stricture may be suspected in these patients if there is difficulty in instrumenting the patient or with a history of recurrent dilation. Contemporary studies have shown difficulty in catheterization, obstructed uroflowmetry pattern, high post-void residue, and urethroscopy as adjuncts in diagnosis, leading the clinician to use more than one diagnostic tests. Low flow rates on uroflowmetry and/or high post-void residue may indicate bladder outlet obstruction due to bladder neck obstruction, detrusor underactivity, or a stricture. Urethral calibration can be a simple but effective way to assess for definite narrowing in the female urethra. Studies in healthy women have reported average calibers of 22F, 23.7±1.9F, and 26F with no correlation between urethral caliber and age (10). The inability to calibrate with 14F can aid in identifying stenosis in the female urethra (11, 12). However, there is no consensus on a definition for female urethral stricture, and other studies have quoted as a criterion for a pathologic urethral caliber ranging from <12F to <20F (8, 13–15). Thus, patients may present with significant LUTS and demonstrate a marginal improvement of symptoms with urethral dilation. However, their urethral caliber can be large enough for a successful calibration or even admit a cystoscope(16). Smith et al. defined FUSD as “a fixed anatomical narrowing of the urethra (<14F) such that the lumen will not accommodate instrumentation without disruption of the urethral mucosal lining”(17). Also, whether having a caliber more than 14F precludes significant BOO is a matter of debate. These patients pose a diagnostic dilemma as to consider them having a dysfunctional voiding pathology or an actual anatomical obstruction.

Video urodynamics enters here to give a clearer picture of the bladder's functional status as it demonstrates both urodynamic and radiographic evidence of bladder outlet obstruction simultaneously along with the corresponding level of blockage. Conditions like detrusor sphincter dyssynergia or dysfunctional voiding may partake urethral stricture features on VCUG and may even accommodate instrument through the urethra. However, features of interrupted flow pattern and altered external sphincter activity during micturition in VUDS help differentiate it from urethral stricture. Hsiao et al. reported Qmax to be significantly lower in anatomic BOO versus dysfunctional voiding. However, there was a considerable overlap in the Qmax between the two groups (18). Patients with dysfunctional voiding might have difficulty initiating a void in public places or might need physical or mental cues to void, such as the sound of running water or the need to ‘deliberately’ relax. Storage symptoms are common and maybe the only presenting symptom.

**Figure 4:**
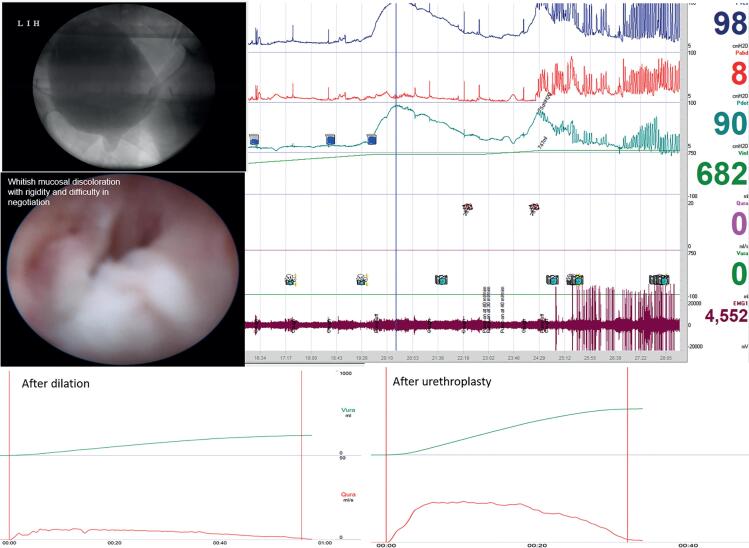
54-year-old lady with IPSS (28) with successful calibration showing VUDS and urethroscopic correlation with pDet@Qmax 90cmH2O, inability to void with proximal urethral ballooning evident during VUDS, stigmata of stricture as a whitish mucosal discoloration and rigidity and poor distensibility on urethroscopy, Qmax after endodilation was ~5mL/s and after urethroplasty ~24mL/s.

Further, VUDS portrays the dynamics of bladder functioning and simultaneously gives a live image of the lower urinary tract and thereby differentiates primary bladder neck obstruction in which high-pressure low flow voids are associated with the impaired opening of the bladder neck will be recorded (18). In addition, coexisting bladder dysfunctions like stress urinary incontinence, detrusor overactivity/underactivity can be identified, which may impact the postoperative outcome of the patient and aid in preoperative counseling of the patient (2, 19). Thus, VUDS should be considered a prerequisite before planning surgery. In our study, 13 out of 16 patients with a caliber of more than 14F along with various urethroscopic abnormalities were found to have significant BOO on VUDS. All of them demonstrated high-pressure low flow voiding with proximal urethral dilation and synchronized activity on EMG (Table-2) (Figures 3 and 4). Spilotros et al. and Mukhtar et al. and have used the presence of urethral ballooning proximal to a portion of urethral narrowing and urodynamic BOO combined with urethrocystoscopy for identifying female urethral stricture (4, 20). Pelvic MRI provides little help in diagnosing non-mass-like urethral pathologies like urethral stricture or periurethral fibrosis (21, 22). Obvious visual narrowing or obliteration on urethroscopy may provide conclusive evidence of urethral stricture. Nevertheless, unlike males, the female urethra is deficient in a thick spongiform layer rather its composed of two muscle coats fused distally, forming a collagenous ring (7). Thus, true narrowing of the lumen might not manifest owing to the deficiency of spongiosum layer rather recurrent submucosal and periurethral scarring might result in a rigid obstruction despite visually near-normal overlying mucosa. Thus, one may hypothesize regarding the singularity of the variable stigmata of stricture on urethroscopy, which was demonstrated in our study (23). Manasa et al. distributed 29 FUSD patients into two categories based on cystoscopy findings. They found a dense anatomical narrowing in 17 patients who underwent surgical management and flimsy strictures in 12 patients looked membranous and broke easily with the beak of the cystoscope. However, all these patients had similar inclusion criteria concerning their IPSS and flow rates, but 12 patients with flimsy stricture were put on self-calibration as the definitive treatment with no comment on the outcomes of this subset of patients (24). In our study, 12 patients had a flimsy or negotiable disease but had a significant obstruction on VUDS. All these patients did extremely well after urethroplasty (Table-2). It is important to emphasize that the treatment of such patients suspected to have urethral stricture disease should be based on the objective criteria of BOO on pressure-flow studies along with calibers it can only be detected on VUDS and not just based on the caliber and flimsiness of stricture encountered. Their symptoms should be accessed through VUDS to document obstruction and critically appraised to rule out dysfunctional components before planning surgical intervention. (Figures 3 and 4).

Urethral dilatation, urethrotomy, intermittent self-catheterization, and substitution urethroplasty are the various options in the management of female urethral stricture. Advocacies in the treatment of FUSD emphasize the good long-term stricture free outcomes after urethroplasty (80-100%) against the short-term improvements via urethral dilation (<50%) (3). Whether dilation is a solution to the problem or a cause by itself is also a matter of debate. Recurrent urethral dilation is associated with trauma leading to inflammation, bleeding, extravasation, etc. augmenting the periurethral fibrosis at the same time maintaining a good caliber (25). It is our belief that after the failure of an initial urethral dilatation, further attempts are less likely to be successful, and this certainly abides by the outcomes in male patients (26). Our study showed significant improvement in mean flow rates after urethroplasty compared to those after endo dilation. We are thus emphasizing that despite providing improved caliber, dilation does not sufficiently relieve a significant obstruction, which probably lies beyond the lumen. Also, recurrent dilation can act as a double-edged sword to further complicate this already complex pathology. Like in male urethral strictures, even in female urethral strictures, many techniques of dorsal and ventral graft/flap urethroplasties have been described (27, 28). However, which technique is to be chosen and when is not yet standardized. We feel it should be better left at the reconstructive surgeon's repose and consideration (28, 29). Our preference has been to use vaginal mucosal graft for reconstruction as it is available near the local site. We chose buccal mucosa graft in three patients where vaginal mucosa was not optimum for reconstruction. However, both types of reconstruction revealed equally good outcomes in our study as underpinned in the available limited literature (3). A dorsal approach can be used for minimizing graft sacculation, providing good mechanical support, and offering a well-vascularized bed for a graft provided by the clitoral cavernosal tissue. It is prudent to stay away from the anterior vaginal wall hence preventing the downward angulation of the urethral meatus, which may impact the direction of the urinary stream. However, injury to the sphincter mechanism, causing incontinence, or to the NVBs of the clitoris, resulting in sexual dysfunction caused by neurosensory impairment, can be worrisome. Nevertheless, studies have found minimal risk of incontinence and, surprisingly, improvement in sexual functions with a dorsal approach in reconstruction (3, 24). Fortunately, we had significant improvements in flow rates and IPSS scores with no evidence of recurrence on 12-month follow-up, embarking upon the efficacy of managing this complex urethral pathology. Except for one patient who had transient stress urinary incontinence after surgery, which had subsided on conservative measures, none of the patients complained of significant leakage and bothersome voiding LUTS after urethroplasty. We feel that the dorsal long segment incision is the key to the good stricture free and leak-free outcomes after urethroplasty in our study.

We advocate a comprehensive and holistic approach towards the diagnosis of female urethral stricture, which includes a high index of suspicion in patients with persistent LUTS despite recurrent dilations, an astute correlation between the clinical aspects of the patient with visual inspection of the narrowing and with a VUDS to objectively confirm or rule out female urethral stricture by following the definition provided by Osman et al., even in the cases of successful calibration of the urethra or a decent flow rate (3). Further we propose, the whitish mucosal discoloration with rigidity and poor distensibility, and difficulty in negotiation on urethroscopy as a vital clue to the diagnosis for FUSD. Also, we believe that significant anatomical obstruction in female urethral stricture disease cannot be solely ruled out based on a good caliber. Further, dilation may provide a good caliber but may still not be sufficient enough in some patients to relieve an obstruction, which probably lies beyond the lumen. This underdiagnosed and undertreated pool of patients who persist in having bothersome LUTS can be identified early via an integrated diagnostic approach for FUSD, as suggested above. These patients with significant obstruction who fail prior dilation as a treatment fair better with an early urethroplasty. To our knowledge, this is the first study to elucidate the clinical and videourodynamics parameters for a prudent and early diagnosis, decision making, and embark upon the management of patients with female urethral stricture, especially even when the patient may be having successful urethral calibration. However, this study is limited by its small sample size and retrospective nature. Further prospective studies are needed to give a more consolidated inference regarding the diagnosis and management of FUSD.

## CONCLUSION

A good caliber of the urethra is not sufficient enough to rule out a significant obstruction due to FUSD. An adept correlation between clinical assessment, urethroscopy findings, and VUDS is vital in identifying FUSD. This also helps in planning surgical management. An early female urethroplasty provides significantly better outcomes in patients who have failed dilation as a treatment.
